# Orbital involvement by spindle cell squamous cell carcinoma: a case report of a rare and aggressive tumor with diagnostic challenges

**DOI:** 10.1186/s12886-026-05166-0

**Published:** 2026-07-31

**Authors:** Gülistan Oyur, Hazal İzol, Fatma Savur

**Affiliations:** 1 Department of Ophthalmology, Başakşehir Çam and Sakura City Hospital, Istanbul, Turkey; 2 Department of Pathology, Başakşehir Çam and Sakura City Hospital, Istanbul, Turkey

**Keywords:** Case report, Immunohistochemistry, Orbital exenteration, Orbital invasion, Spindle cell squamous cell carcinoma

## Abstract

**Background:**

Spindle cell squamous cell carcinoma (SCSCC) is a rare and poorly differentiated variant of squamous cell carcinoma that may mimic sarcoma, melanoma, and other spindle cell malignancies. Orbital involvement is exceptionally uncommon and may pose substantial diagnostic and therapeutic challenges, particularly when overt squamous differentiation is absent.

**Case presentation:**

A 60-year-old woman with a history of left ocular evisceration for a blind painful eye presented six months later with rapidly progressive painful swelling of the left lower eyelid and medial canthus. Orbital magnetic resonance imaging demonstrated an infiltrative, heterogeneously enhancing orbital mass, and positron emission tomography-computed tomography showed hypermetabolic uptake in the orbital lesion with additional ipsilateral cervical and parotid-region foci. The patient underwent orbital exenteration combined with radical neck dissection and radical parotidectomy. Histopathologic examination revealed a malignant spindle cell neoplasm with marked pleomorphism and frequent mitotic activity, without definite keratinization or overt squamous differentiation. Immunohistochemical staining showed strong positivity for pancytokeratin AE1/AE3, CK OSCAR, and CK8/18, supporting epithelial differentiation and a diagnosis most consistent with spindle cell squamous cell carcinoma. Despite adjuvant radiotherapy and systemic chemotherapy, metastatic disease developed within three months, and the patient died six months after orbital exenteration.

**Conclusions:**

This case highlights the diagnostic difficulty and aggressive clinical behavior of orbital spindle cell malignancies with features most consistent with spindle cell squamous cell carcinoma. Broad immunohistochemical evaluation, careful clinicopathological correlation, comprehensive staging, and early multidisciplinary management are essential in such rare and high-risk tumors.

## Introduction

Spindle cell squamous cell carcinoma (SCSCC) is an uncommon and poorly differentiated variant of squamous cell carcinoma that accounts for less than 3% of head and neck SCC [1, 2]. Histologically, it exhibits a biphasic architecture consisting of malignant epithelial and spindle cell (sarcomatoid) components and often mimics true sarcomas or other spindle cell neoplasms. Compared to conventional SCC, this variant demonstrates a more fulminant clinical course, characterized by rapid local invasion and early metastatic spread [1].

SCSCC is most commonly seen in the upper respiratory tract and digestive system, namely the larynx, oral cavity, and pharynx, while ocular adnexal or orbital involvement is exceedingly rare, with a limited number of cases documented in the literature to date [2, 3]. Such uncommon conditions pose significant diagnostic challenges due to their morphological overlap with sarcomas, melanomas, and atypical fibroxanthomas and often require extensive immunohistochemical profiling for confirmation. Furthermore, these tumors have a poor prognosis, with high rates of local recurrence and distant metastasis reported even after intensive multimodal therapy [1, 4].

Given their rare and aggressive nature, early diagnosis of SCSCC is crucial for timely intervention and can influence surgical planning and adjuvant treatment decisions. In this report, we present an unusual case of SCSCC with orbital invasion and discuss the relevant literature to highlight diagnostic challenges and treatment approaches.

## Case presentation

A 60-year-old woman with poorly controlled diabetes mellitus and a history of neovascular glaucoma underwent evisceration of the left eye for a blind painful eye one year after pars plana vitrectomy performed for proliferative diabetic retinopathy. Six months postoperatively, she presented with rapidly progressive painful swelling of the left lower eyelid and medial canthus. She had no history of periocular malignancy or trauma.

**Fig. 1:**
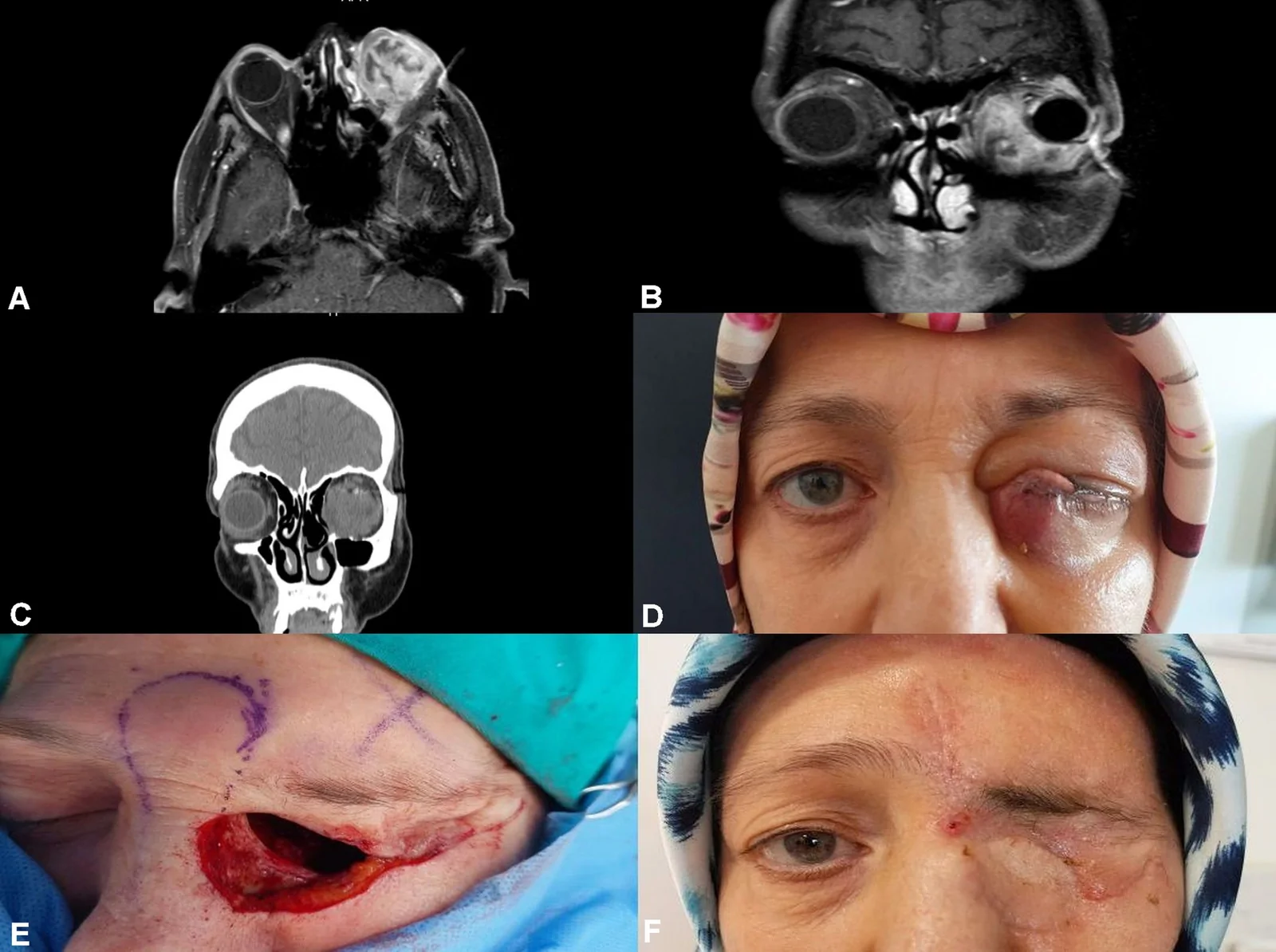
Orbital imaging findings, clinical presentation, and surgical management. (**A**) Axial contrast-enhanced magnetic resonance imaging demonstrating a heterogeneously enhancing mass involving the left orbit and periocular soft tissues. (**B**) Coronal magnetic resonance imaging showing superior and anterior extension of the lesion. (**C**) Coronal computed tomography image demonstrating the left orbital mass with postsurgical changes related to prior evisceration. (**D**) Preoperative clinical photograph showing a bulky protruding mass involving the left periocular region. (**E**) Intraoperative view during orbital exenteration. (**F**) Early postoperative appearance following orbital exenteration

Clinical examination revealed a firm, ill‑defined orbital mass without regional lymphadenopathy. Orbital magnetic resonance imaging (MRI) demonstrated a heterogeneously contrast‑enhancing, infiltrative soft tissue mass occupying the eviscerated orbital space and extending into the preseptal and postseptal spaces, with focal thinning of the orbital walls but no intracranial extension (Fig. 1).

**Fig. 2:**
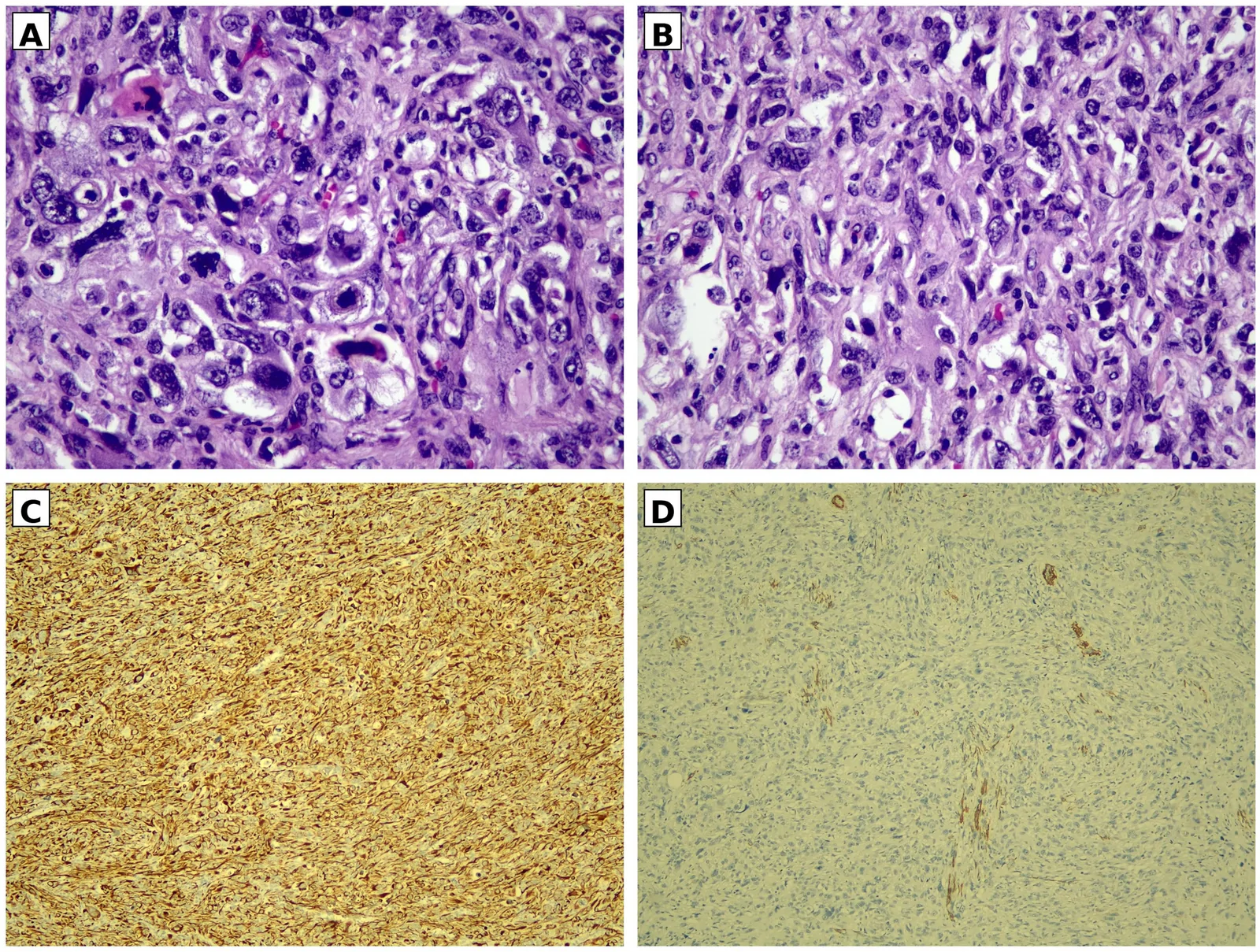
Histopathologic and immunohistochemical findings of the orbital tumor. (**A**) Higher-magnification hematoxylin and eosin image at ×40 showing marked nuclear pleomorphism, hyperchromasia, and tumor cell atypia. (**B**) Higher-magnification hematoxylin and eosin image at ×40 demonstrating spindle-shaped tumor cells. (**C**) Serial/adjacent section showing diffuse pancytokeratin AE1/AE3 positivity at ×10, supporting epithelial differentiation. (**D**) Alpha-smooth muscle actin (α-SMA) immunostaining at ×10 showing negative staining in tumor cells, helping to exclude smooth muscle/myogenic differentiation

Preoperative PET-CT demonstrated an intensely hypermetabolic left orbital mass with additional hypermetabolic foci in the ipsilateral cervical lymph nodes and parotid region, raising concern for regional metastatic spread. Given the extensive infiltrative nature of the lesion and concern for regional involvement, the patient underwent orbital exenteration together with left radical neck dissection and left radical parotidectomy in collaboration with the otorhinolaryngology–head and neck surgery team. Postoperatively, adjuvant radiotherapy and systemic chemotherapy were administered.

Histopathologic examination of the exenterated specimen revealed a malignant spindle cell neoplasm arranged in fascicles with marked nuclear pleomorphism, hyperchromasia, and frequent mitotic figures. No areas of keratinization or overt squamous differentiation were identified, contributing to the diagnostic challenge. Immunohistochemical analysis showed strong pancytokeratin AE1/AE3 and CD10 expression, with additional positivity for CK OSCAR and CK8/18, while epithelial membrane antigen (EMA), p40, and alpha-smooth muscle actin (α-SMA) were negative.

In conjunction with the morphologic features, the broad-spectrum cytokeratin positivity supported a diagnosis most consistent with spindle cell squamous cell carcinoma. Representative histopathologic and immunohistochemical findings are shown in Fig. 2.

Postoperatively, the patient received adjuvant orbital radiotherapy and systemic chemotherapy with cisplatin and docetaxel. Follow‑up PET‑CT at three months revealed new metastatic deposits in the cervical lymph nodes and vertebral bodies (C6–C7), indicating rapid disease progression and poor prognosis. Despite continued systemic treatment, the patient died six months after orbital exenteration due to progressive metastatic disease.

## Discussion

SCSCC with orbital involvement remains an exceptionally rare entity, and the available literature is largely limited to isolated case reports and small case series [1, 4, 5]. Most previously reported periocular or orbital cases have been associated with cutaneous squamous cell carcinoma showing secondary orbital invasion, whereas primary conjunctival, socket mucosal, or de novo orbital presentations appear to be far less common [2, 3] In this context, the present case is clinically relevant not only because of its rarity, but also because it illustrates several overlapping challenges that are particularly important in orbital practice: development of a spindle cell malignancy in a previously operated socket with uncertain primary origin, absence of overt squamous differentiation on routine histology, negativity for conventional squamous markers such as p40 and EMA, potentially misleading CD10 expression, and rapid metastatic progression despite aggressive multimodal treatment. The case therefore contributes to the limited literature by illustrating the diagnostic uncertainty, possible sites of origin, and aggressive clinical course that may accompany orbital spindle cell malignancies [2–4].

The diagnostic interpretation in the present case should be made with appropriate caution. The diagnostic complexity of SCSCC largely arises from its biphasic morphology, comprising both squamous epithelial and sarcomatoid spindle cell components [1, 5]. Histopathologically, these tumors may mimic other high-grade spindle cell neoplasms, including sarcomatoid carcinoma, atypical fibroxanthoma, pleomorphic dermal sarcoma, malignant melanoma, malignant peripheral nerve sheath tumor, and true spindle cell sarcomas, thereby necessitating a comprehensive immunohistochemical panel for accurate classification [6, 7]. In our patient, diffuse CD10 expression initially raised suspicion for atypical fibroxanthoma or pleomorphic dermal sarcoma. CD10 expression may be seen in spindle cell squamous cell carcinoma, but it is not specific and should be interpreted in conjunction with morphology and broad-spectrum cytokeratin positivity, particularly in the distinction from atypical fibroxanthoma and other spindle cell malignancies [6]. The tumor cells demonstrated strong and diffuse positivity for broad-spectrum cytokeratins, including pancytokeratin AE1/AE3, CK OSCAR, and CK8/18, supporting epithelial differentiation despite the absence of overt squamous differentiation and classic squamous markers such as p40 and EMA [5, 7]. Therefore, the overall findings were considered most consistent with rather than an unequivocally confirmed classic SCSCC. This diagnostic nuance underscores the importance of integrating morphological assessment with a comprehensive immunohistochemical profile and the full clinicopathological context rather than relying on a single marker.

The primary site of origin in the present case remains uncertain and represents an important clinicopathological issue. Several possibilities should be considered, including an occult periocular cutaneous primary with secondary orbital extension, origin from conjunctival or socket mucosal epithelium, or development from residual periocular epithelial structures within the previously operated socket. Although the initial evisceration specimen reportedly showed no evidence of malignancy, the possibility of an unsampled microscopic neoplastic focus cannot be completely excluded. Conversely, the six-month interval between evisceration and the appearance of the orbital mass may also support the possibility of an independently arising tumor rather than direct persistence of a pre-existing lesion. Because the presumed site of origin may influence surgical margin assessment, regional staging, and multidisciplinary treatment planning, careful clinicopathological correlation and thorough periocular examination are essential in similar cases [2–4].

In addition to histopathologic assessment, cross-sectional imaging and systemic staging are essential in orbital SCSCC because these tumors may show infiltrative local spread, regional nodal involvement, and early distant metastasis [2–4]. In the present case, MRI demonstrated an irregular infiltrative orbital mass involving the eviscerated socket and adjacent periocular soft tissues, with focal thinning of the adjacent orbital walls but no definite intracranial extension. These radiologic findings were concordant with the high-grade spindle cell morphology and locally aggressive behavior observed histopathologically. PET-CT further demonstrated intense uptake within the orbital lesion and additional ipsilateral cervical and parotid-region hypermetabolic foci, raising concern for regional metastatic spread and supporting the decision to perform combined orbital and head-and-neck surgical management.

Management of orbital SCSCC is not standardized because of the rarity of the disease and the lack of tumor-specific treatment guidelines. In reported cases, treatment has generally been individualized according to the extent of local invasion, regional nodal disease, margin status, and systemic involvement, with radical surgical excision, often including orbital exenteration, serving as the mainstay for locally advanced disease [2–4]. In the present case, the infiltrative orbital mass, suspected regional nodal and parotid involvement on PET-CT, and aggressive histopathologic features justified a combined surgical approach consisting of orbital exenteration, radical neck dissection, and radical parotidectomy. Adjuvant radiotherapy was administered for local–regional control, and systemic chemotherapy with cisplatin and docetaxel was initiated by the medical oncology team as part of multidisciplinary oncologic management, given the aggressive squamous carcinoma biology and concern for regional or systemic spread. Nevertheless, the development of cervical and vertebral metastases within three months, followed by death six months after orbital exenteration despite continued systemic treatment, highlights the limited effectiveness of conventional multimodal therapy in some advanced cases and is consistent with the poor outcomes reported for metastatic ocular adnexal SCSCC and head-and-neck sarcomatoid carcinomas [4, 8].

The potential role of immune checkpoint inhibitors also deserves consideration. Although evidence specific to orbital SCSCC remains extremely limited, advanced cutaneous squamous cell carcinoma and related non-melanomatous spindle cell neoplasms may respond to PD-1/PD-L1–directed immunotherapy, particularly in tumors with ultraviolet-related mutational signatures or high tumor mutational burden [9]. In this regard, molecular profiling, including assessment of PD-L1 expression, tumor mutational burden, and other actionable alterations, may be valuable in future cases, especially when conventional surgery, radiotherapy, and chemotherapy are unlikely to achieve durable disease control. However, the applicability of immunotherapy to orbital SCSCC remains uncertain and should be evaluated within a multidisciplinary oncologic framework.

This report has several limitations. It describes a single case, and therefore no generalizable conclusions regarding prognosis or treatment efficacy can be made. The follow-up period was limited by the rapid clinical progression and death of the patient six months after orbital exenteration. In addition, the diagnosis was based on morphology and immunohistochemical evidence of epithelial differentiation, without molecular confirmation. PD-L1 expression, tumor mutational burden, and other molecular markers were not evaluated. Finally, the primary site of origin could not be determined with certainty. These limitations should be considered when interpreting the diagnostic and therapeutic implications of the case.

Overall, this case underscores the need for heightened clinical suspicion and prompt histopathological evaluation in atypical orbital masses to ensure early and accurate diagnosis. Awareness of this rare variant, combined with a systematic immunohistochemical approach, may aid clinicians in optimizing management and counseling regarding prognosis.

## Conclusion

SCSCC with orbital involvement is a rare and aggressive malignancy that may pose substantial diagnostic difficulty, particularly in the absence of classic squamous differentiation. This case highlights the importance of careful histopathologic and immunohistochemical evaluation, multidisciplinary surgical planning, and close oncologic follow-up in managing such unusual and high-risk tumors.

## Data Availability

All data supporting the findings of this case report are included in the article and are publicly available as part of this published article.
